# Supplementary material for: Transcriptome Analysis of Improved Wool Production in Skin-Specific Transgenic Sheep Overexpressing Ovine β-Catenin
Source: Int J Mol Sci. 2019 Jan 31;20(3):620. doi: 10.3390/ijms20030620 (PMC6387261; doi:10.3390/ijms20030620)
Supplement: Supplementary file 1 [file ijms-20-00620-s001.zip › ijms-402609-Figure S1.docx]

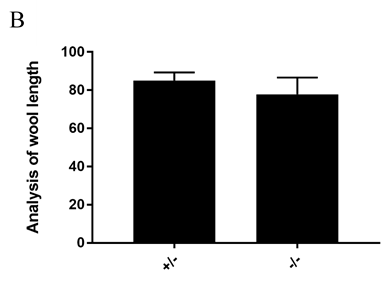

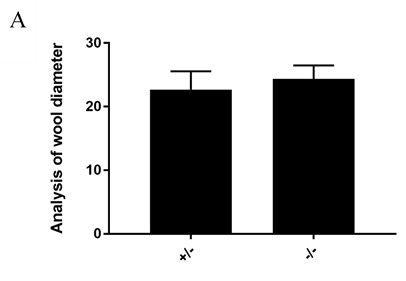


Figure S1: Analysis of wool length and diameter. A. The wool diameter of F1 transgenic sheep and wild type sibling were measured by microscope projector. B. The wool length of F1 transgenic sheep and wild type sibling were measured by Array diagram method. Paired student’s t test was used to determine the significance of wool diameter and length between transgenic sheep and wild type sibling.
